# Mr. Watt, on Vaccine

**Published:** 1801-05

**Authors:** R. Watt

**Affiliations:** Paisley


### *To the Editors of the Medical and Phyfical Journal.*

Gentlemen,

If you think the following cafes deferve a place in your very ufeful Publication, you are at liberty to infert them. The firft proves that the Small-pox do not prevent the Vaccine Contagion from taking its ufual effect, if properly applied to the body. The fecond proves that the two difeafes may exift at the fame time in the fame perfon, and yet continue perfectly diftinct.

Case First.—In the beginning of February laft, I inoculated M. W. a girl about five years of age, with Vaccine Matter on both arms. After fhe had gone through the ufual ftages of the difeafe, about the eleventh or twelfth day I opened the puftules, and was extracting matter from them for the purpofe of inoculating others. Her uncle, Mr. W. a man about twenty-fix years of age, was looking on, and appeared to be very much taken up in examining the appearance and confiftence of the matter; and I obferved him frequently touching it with his finger.

On the feventh day after this, I was called to examine his noftrils, which appeared very much excoriated and inflamed, in confequence of a fevere cold he had received a few weeks before: The falivary glands too, on both fides, had begun to fwell, and were become exceedingly painful. He attributed this to a frefh cold he had received a few days ago, and which appeared to me extremely probable. I ordered leeches to be applied to the inflamed glands, and directed him not to expofe himfelf again to the cold till he had fairly recovered.

On the eighth day the fwelling in the glands continued, but the pain was confiderably relieved fince the bleeding. The noftrils were more inflamed, and the nofe itfelf was fwelled to a very great fize, and extremely painful: I ordered leeches to be applied to it, which gave him confiderable relief.

On the ninth day the pain in the nofe had returned, and the fwelling continued to increafe. The patient was exceedingly alarmed alarmed, and infifted to know what I thought was the matter with his nofe: He faid he believed it was a cancer, for the nofe was often fubject to that difeafe; I affured him, from its appearance, and the rapid progrefs it had made, that I was convinced that it was not a cancer. On examining carefully his noftrils, I could perceive fomething in each of them that very nearly refembled a Vaccine puftule: The nearer I examined it I found the refemblance ftill more complete. I afked him whether or not he thought any of the Cow-pox matter could poffibly have got into his noftrils, as I recollected his handling fome of it when I took it from his niece's arms. He faid it was very poffible, for at that time he carried a fmall box of ointment in his pocket, with which he ufed frequently to anoint his noftrils; and that he might probably have done this with the fame finger with which he had touched the Vaccine Matter.

This account confirmed my fufpicions, and removed my fears; for I muft confefs I was confiderably alarmed at fuch a fingular phenomenon, fo widely different from any thing I had ever feen. I told him not to be afraid, he had only inoculated himfelf with the Cow-pox, and would foon be better. In three or four days more the puftules were completely formed, and the difeafe at laft terminated in the ufual way. In two weeks after the noftrils were completely whole; and what is fingular, for feveral years paft, Mr. W. in the fpring feafon was much afflicted with fore noftrils; and this year, prior to the inoculation, they appeared to be worfe than ever; but fince that time they have been perfectly found, and the fkin ftronger than it has been for feveral years paft. May not the infertion of Vaccine Matter into fome inveterate ulcers produce permanent cure?

Mr. W. had the Small-pox, without inoculation, about twenty years ago, of which fome flight veftiges ftill remain.

Case Second.—On the 4th of February laft, Mrs. B. applied to me to inoculate her child, who was then about five months old. She faid fhe was very much alarmed, for the Small-pox a few days ago had appeared in the neighbourhood where fhe lived, and two children had died of them. At this time I happened to be out of matter, and could not procure any for three days. On the 7th of February the child was inoculated in both arms, with frefh matter taken immediately from the arm of another child and applied to her.

I faw her again on the fourth day after the inoculation, when fhe appeared to be in a very high fever, and had continued fo for two days. I fufpected, as fhe was near to patients who had the the Small-pox, that ſhe might probably have caught the infection, prior to the inoculation with the Cow-pox. I examined her body carefully, but could perceive no eruptions that could confirm my ſuſpicions.

On the fifth day, however, the fever had begun to abate, and the eruption appeared: She had about a hundred diſtinct puſtules on her whole body; theſe continued for about ſeven or eight days, and then dryed and ſcaled off; during which time ſhe was in good health: the Vaccine Inoculation had alſo taken effect, and both arms inflamed, and went through the ſame ſtages as if no other diſeaſe had exiſted. On the eleventh day after the inoculation, I opened the puſtules, and extracted a quantity of matter from them, with which I inoculated ſeveral others, who all took the Cow-pox in the uſual way, without any other eruption whatever.

I made enquiry in the neighbourhood of the place where Mrs. B. lived, concerning the patients from whom her child had caught the infection, and I found that all of them had been extremely ill. That out of eleven, five had died, and the other ſix had eſcaped very narrowly. It was Mrs. B's fourth child; her two firſt had died of the Small-pox; they were a year old: the third is living, but very much marked by them.

May not the inoculating with Vaccine Matter in caſes where we ſuſpect that the patients are infected with the Small-pox, render that diſeaſe more mild than it would otherwiſe be?

I am, &c.

*Paisley, March* 27, 1801. R. WATT.

